# Predicting mortality in acutely hospitalized older patients: a retrospective cohort study

**DOI:** 10.1007/s11739-015-1381-7

**Published:** 2016-01-29

**Authors:** Jelle de Gelder, Jacinta A. Lucke, Noor Heim, Antonius J. M. de Craen, Shantaily D. Lourens, Ewout W. Steyerberg, Bas de Groot, Anne J. Fogteloo, Gerard J. Blauw, Simon P. Mooijaart

**Affiliations:** Department of Gerontology and Geriatrics, Leiden University Medical Center, PO Box 9600, 2300 RC Leiden, The Netherlands; Department of Emergency Medicine, Leiden University Medical Center, Leiden, The Netherlands; Department of Public Health, Erasmus MC, Rotterdam, The Netherlands; Department of Internal Medicine, Section Acute Care, Leiden University Medical Center, Leiden, The Netherlands; Institute of Evidence-Based Medicine in Old Age, IEMO, Leiden, The Netherlands

**Keywords:** Acute hospitalization, Prediction, Mortality, Older adults, Elderly

## Abstract

Acutely hospitalized older patients have an increased risk of mortality, but at the moment of presentation this risk is difficult to assess. Early identification of patients at high risk might increase the awareness of the physician, and enable tailored decision-making. Existing screening instruments mainly use either geriatric factors or severity of disease for prognostication. Predictive performance of these instruments is moderate, which hampers successive interventions. We conducted a retrospective cohort study among all patients aged 70 years and over who were acutely hospitalized in the Acute Medical Unit of the Leiden University Medical Center, the Netherlands in 2012. We developed a prediction model for 90-day mortality that combines vital signs and laboratory test results reflecting severity of disease with geriatric factors, represented by comorbidities and number of medications. Among 517 patients, 94 patients (18.2 %) died within 90 days after admission. Six predictors of mortality were included in a model for mortality: oxygen saturation, Charlson comorbidity index, thrombocytes, urea, C-reactive protein and non-fasting glucose. The prediction model performs satisfactorily with an 0.738 (0.667–0.798). Using this model, 53 % of the patients in the highest risk decile (*N* = 51) were deceased within 90 days. In conclusion, we are able to predict 90-day mortality in acutely hospitalized older patients using a model with directly available clinical data describing disease severity and geriatric factors. After further validation, such a model might be used in clinical decision making in older patients.

**Electronic supplementary material:**

The online version of this article (doi:10.1007/s11739-015-1381-7) contains supplementary material, which is available to authorized users.

## Introduction

Acute medical illness in older adults is a serious contributor to deterioration [1]. Within 90 days after hospitalization, approximately 20 % will die [2, 3]. At the time of admission it is difficult to determine who is at highest risk. Visualizing the individual risk in an early phase of hospitalization might increase the awareness of the physician, and enable tailored decision-making for the older patient, although these interventions may not primarily be aimed at reducing mortality. A high risk of mortality may reflect overall vulnerability, which preventive interventions may be aimed at, or conversely by usefully initiating palliative care.

Screening models to identify older patients at risk of mortality have been developed and evaluated [4, 5]. These models mainly use either geriatric factors [5, 6] or severity of disease [7, 8]. In these models, scores are assigned to the predictors, which lead to a total score with a cut-off point for high-risk patients. Predictive performance using the cut-off point shows relatively high sensitivity and low specificity, resulting in high numbers of false positives. As a consequence, large-scale implementation of these models in daily care hampers successive interventions [6]. A combination of routine clinical parameters, which reflect the severity of disease, in combination with geriatric factors might improve accuracy and feasibility in daily care.

In the present retrospective cohort study we developed a prediction model for 90-day mortality. We collected clinical parameters of all hospitalized older patients of the acute medical unit in 2012. Vital signs and laboratory results reflect severity of disease with geriatric factors, represented by comorbidities and number of medications. We selected variables that are available in a very early phase of hospitalization to enable in-hospital interventions.

## Methods

### Study design and setting

We performed a retrospective follow-up study among all patients aged 70 years and over who were acutely hospitalized on the acute medical unit (AMU) of the Leiden University Medical Center (LUMC), the Netherlands in 2012. Any following individual admission in the study period, independent of the reason, and patients with palliative care who were expected to die in a few days were excluded. The AMU is a 13-bed ward particularly focussed on acute admissions, mainly from the Emergency Department. The population is characterized by hemodynamically stable patients in the fields of internal medicine, surgery, pulmonary diseases and gastroenterology. The medical ethics committee of the LUMC waived the necessity for formal approval of the present study, as all data were available from standard care.

### Predictors

We selected potential predictors of 90-day mortality from the clinical parameters available at the moment of hospitalization on the AMU. These parameters reflect severity of disease, including vital signs and laboratory results, and underlying level of vulnerability, including comorbidity and number of medications used at home. A predictor was eligible if it fulfilled the following criteria: (1) it was available in the medical records for retrospective analysis; (2) available to the physician within 24 h after admission and (3) assumed to have a relationship to the outcome based on clinical reasoning by three medical doctors and (4) was already being measured routinely to enhance in future implementation, with a maximum of 15 % missing values of each predictor. Multiple imputation techniques were used to compute the missing predictors [9]. First measured vital signs after hospitalization and first known in-hospital laboratory results were extracted from the electronic patient records (Chipsoft-EZIS^®^, version 5.2, 2006–2014). Existing comorbidities and medications used at home were obtained manually from the patient records, where medication was reported as part of routine clinical practice. Usually, the physician will first ask the patient at the moment of hospitalization for comorbidities and medication use. If necessary, the information will be verified with the general practitioner or pharmacy.

Vital signs were assessed by the nurse directly after admission and consisted of systolic and diastolic blood pressure, heart rate, respiratory rate, oxygen saturation and body temperature. First known in-hospital laboratory results within 24 h after presentation were extracted and consisted of: sodium (mmol/L), potassium (mmol/L), urea (mmol/L), eGFR (estimated glomular filtration rate, calculated by the modification of diet in renal disease (MDRD) equation, ml/min/1.73 m^2^), leukocytes (×10^9^/L), thrombocytes (×10^9^/L), C-reactive protein (mg/L), non-fasted glucose (mmol/L) and haemoglobin (mmol/L). Comorbidity was evaluated with the Charlson comorbidity index (CCI). It incorporates weighted scores for 19 medical conditions, increasing from 1 to 6 with severity. The CCI is a frequently used instrument by researchers, and has been validated to predict 1-year mortality [10, 11]. The number of different medications at home was recorded according to their pharmacological sub classification. Medications of the same subgroup count as one drug, and combined medications of two different pharmacological sub-classifications were considered as two different drugs. Topical and ‘as required’ medications were excluded because of the unreliable registration rate of the physicians and the absence of information whether the patient actually used it. If recorded in the medical records, over-the-counter medications were included when patients used it on regular base.

### Outcome

The primary endpoint was mortality within 90 days after hospital admission. Mortality dates were assessed from the Dutch municipality records.

### Coding predictors

We aimed to develop a model with a high positive predictive value (PPV) to enable targeted interventions, and therefore the model should have high specificity. Because a model based on a risk score derived from clinical cut-off values is easier to implement in clinical practice than a model based on computations with continuous variables, we started to dichotomize continuous predictors by using the ranges of clinical reference categories. A stricter clinically relevant cut-off point was chosen in cases when specificity on 90-day mortality was lower than fifty percent. However, dichotomizing may lead to loss of information, reduction in power and uncertainly in defining the optimal cutpoint [12]. In a sensitivity analysis, we repeated the same analyses with preservation of continuous predictors to compare discriminative performance.

### Statistical/data analysis

Descriptive baseline characteristics were expressed in percentages, means with standard deviations and medians with interquartile ranges. A Kaplan–Meier curve was used to show cumulative mortality of patients after admission. Binary logistic regression techniques for both dichotomised and continuous data were used for uni- and multivariable analysis on mortality (no censoring occurred). The prediction model was derived via backward elimination with Akaike’s Information Criterion for candidate predictors. With this technique the most significant predictors remain in the model, while “noise” is reduced by eliminating predictors that are not statistically significant. Discriminative performance of the different models was assessed with the area under the receiver operating characteristic curve (AUC).The model was internal validated with 500 bootstrap samples, where we repeated the backward elimination procedure. With this method internal validity is tested by drawing 500 different population samples from the original sample. The test reflects how robust the findings are when slight changes are made to the population, and was presented with the internal validated AUC. The formula 1/(1 + exp^(−linear predictor)^)was applied to determine the individual risk on 90-day mortality [13]. Performance of the final model is shown with the sensitivity, specificity, positive predictive value (PPV), negative predictive value (NPV), positive likelihood ratio and negative likelihood ratio.

In clinical practice, cut-off points are often used, e.g., to interpret laboratory results. In prediction dichotomizing continuous predictors is arguable, because of a loss of information [12]. When using cut-off points, laboratory results just outside the reference range are considered the same risk as more extreme results, which diminishes the power of the model. In a sensitivity analyses we treated predictors as continuous variables to compare discrimination. First outlying observations were truncated to 5 and 95 % by means of winsorization [13]. Second restricted cubic spline techniques with three knots were applied to continuous predictors in a binary regression model, and discrimination was analysed by calculation the AUC. The level of significance was set at *P* < 0.05. Statistical analyses were performed using IBM SPSS Statistics package (version 20) and R version 3.1.1.

## Results

In 2012, 606 older patients were acutely hospitalized to the acute medical unit (AMU) of our hospital. By excluding 86 subsequent admissions and 3 moribund patients, a final cohort of 517 patients was available for final analysis.

The baseline characteristics of the cohort are described in Table 1. The mean age was 78.3 years, 269 (52.0 %) patients were male, 467 (90.3 %) were admitted via the Emergency Department and 367 (71.0 %) were primary treated under the responsibility of one of the medical specialities (internal medicine, surgery or pulmonary diseases). Mean laboratory results were within the normal range or slightly below or above these thresholds. The median number of comorbidities was 2 (IQR 1-4), and median number of medications used at home was 7 (IQR 4-11).

**Table 1:** Baseline characteristics of the study population

Characteristics	*N* = 517
Male, *n* (%)	269 (52.0 %)
Age, mean (SD)	78.3 (6.2)
Admitted from, *n* (%)	
Emergency department	467 (90.3 % )
Outpatient clinic	42 (8.1 %)
Other	8 (1.6 %)
Clinical specialism, *n* (%)	
Internal medicine	367 (71.0 %)
Surgery	74 (14.3 %)
Pulmonary diseases	34 (6.6 %)
Other	42 (8.1 %)
**Severity of disease**	
Vital parameters^a^	
Oxygen saturation (%), median (IQR)	98 (96–99)
Systolic blood pressure (mm Hg), mean (SD)	132.9 (23.3)
Diastolic blood pressure (mm Hg), mean (SD)	67.6 (13.9)
Heart rate (/min), mean (SD)	83.2 (16.6)
Laboratory results	
Sodium (mmol/L), mean (SD)	138.6 (5.4)
Potassium (mmol/L), mean (SD)	4.3 (0.7)
Urea (mmol/L), median (IQR)	9.4 (6.7–14.7)
eGFR (ml/min/1.73 m^2^), mean (SD)	64.8 (34.5)
Leukocytes (×10^9^/L), mean (SD)	11.3 (5.3)
Thrombocytes (×10^9^/L), mean (SD)	241 (119)
C-reactive protein (mg/L), median (IQR)	41 (8–110)
Non-fasted glucose (mmol/L), mean (SD)	8.1 (3.6)
Haemoglobin (mmol/L), mean (SD)	7.6 (1.5)
**Geriatric factors**	
Charlson comorbidity index, median (IQR)^b^	2 (1–4)
Number of medications, median (IQR)	7 (4–11)

*eGFR* estimated glomerular filtration rate; *SD* standard deviation, *IQR* inter quartile range

^a^Respiratory rate and body temperature were excluded from further analysis, because the measurement was not performed or noted in >50 %

^b^Incorporates weighted scores for 19 medical conditions, higher scores indicating worse history of disease

Supplemental Table 1 gives an overview of categories of the dichotomization process. Missing values, up to 11 % in thrombocytes, were imputed. Most reference ranges reflect clinical normal ranges, except that we chose different rounded cut-offs for systolic blood pressure (<200 mmHg), urea (<15.0 mmol/L) and c-reactive protein (CRP, <100 mg/L), leukocytes (<13 × 10^9^/L), eGFR (>30 ml/min/1.73 m^2^) and haemoglobin (>6.5 mmol/L for females and >7.5 mmol/L for males) to reflect more specific measures of disease severity.

After 90 days, 94 patients (18.2 %) had died (supplemental Fig. 1). In Table 2, results of the univariate analyses and the performance of all individual predictors are shown. Oxygen saturation, heart rate, Charlson comorbidity index (CCI), thrombocytes, urea, potassium and CRP outside the reference range are statistically significantly associated with 90-day mortality. In contrast, non-fasted glucose and creatinine clearance outside the reference range prove to have a protective effect. Age and gender show no association with 90-day mortality. Best performance of a single variable is the CCI. A score of 5 or higher (*N* = 91) yields a positive predictive value (PPV) of 0.37 and area under the curve (AUC) of 0.61.

**Table 2:** Univariate associations and the performance of single predictors of 90-day mortality in acutely hospitalized older patients

	Univariate		Performance					
	Number (%)^a^	OR	95 % CI	Sens	Spec	PPV	NPV	AUC
Age (per 5 years increase)	–	1.05	0.88–1.25	–	–	–	–	0.52
Male	269 (52)	1.38	0.88–2.17	0.59	0.49	0.20	0.84	0.54
Saturation	108 (21)	2.21	1.35–3.63	0.33	0.82	0.29	0.85	0.57
Systolic blood pressure	39 (8)	1.39	0.64–3.03	0.10	0.93	0.23	0.82	0.51
Diastolic blood pressure	206 (40)	1.21	0.77–1.90	0.44	0.61	0.20	0.83	0.52
Heart rate	136 (26)	1.68	1.04–2.71	0.35	0.76	0.24	0.84	0.55
Charlson comorbidity index^b^	91 (18)	3.64	2.20–6.03	0.36	0.87	0.37	0.86	0.61
Number of medications	153 (30)	1.53	0.96–2.45	0.37	0.72	0.23	0.84	0.55
Thrombocytes	139 (27)	2.16	1.35–3.46	0.40	0.76	0.27	0.85	0.58
Urea	126 (24)	2.44	1.52–3.92	0.39	0.79	0.29	0.85	0.59
Leukocytes	169 (33)	1.43	0.90–2.27	0.39	0.69	0.22	0.84	0.54
Sodium	162 (31)	1.16	0.72–1.87	0.34	0.69	0.20	0.83	0.52
Potassium	134 (26)	1.73	1.07–2.78	0.35	0.76	0.25	0.84	0.56
Haemoglobin	161 (31)	1.57	0.98–2.49	0.39	0.71	0.23	0.84	0.55
C-reactive protein	141 (27)	1.77	1.11–2.85	0.37	0.75	0.25	0.84	0.56
Non-fasted glucose	361 (70)	0.51	0.32–0.81	0.43	0.73	0.26	0.85	0.58
eGFR	76 (15)	0.48	0.28–0.84	0.23	0.87	0.29	0.84	0.55

*eGFR* estimated glomerular filtration rate

^a^Number represents number of patients outside reference category

^b^Incorporates weighted scores for 19 medical conditions, higher scores indicating worse history of disease

Results of the multivariable and final model are displayed in Table 3. A backward selection procedure results in a model of six predictors including oxygen saturation, CCI, thrombocytes, urea, CRP and non-fasted glucose. The area under the curve (AUC) is 0.738 (95 %CI 0.967–0.798) and decreases to 0.724 after internal validation.

**Table 3:** Multivariate and final model of predictors of 90-day mortality in acute hospitalized older patients

	Multivariate		Final model			
	OR	95 % CI	*β*	OR	95 % CI	*P* value
Age (per 5 years)	1.20	0.97–1.47				
Male	1.34	0.79–2.25				
Saturation	2.32	1.34–4.03	0.862	2.37	1.39–4.05	0.002
Systolic blood pressure	0.96	0.38–2.45				
Diastolic blood pressure	1.02	0.60–1.72				
Heart rate	1.58	0.93–2.70				
Charlson comorbidity index^a^	3.45	1.95–6.10	1.201	3.32	1.94–5.70	<0.001
Number of drugs	1.43	0.79–2.58				
Thrombocytes	2.10	1.24–3.56	0.774	2.17	1.30–3.62	0.003
Urea	1.90	0.98–3.66	0.706	2.03	1.21–3.41	0.008
Leukocytes	1.24	0.73–2.11				
Sodium	1.08	0.64–1.82				
Potassium	1.38	0.80–2.40				
Haemoglobin	1.06	0.62–1.81				
C-reactive protein	1.65	0.96–2.81	0.588	1.80	1.08–2.99	0.023
Non-fasted glucose	0.44	0.26–0.73	−0.791	0.45	0.28–0.75	0.002
eGFR	1.24	0.57–2.69				
Intercept			−2.127			
AUC (95 % CI)			0.738 (0.678–0.798)			
Internal validated AUC			0.724			

*eGFR* estimated glomerular filtration rate, *AUC* area under curve, *Internal validated AUC* the obtained AUC after bootstrapping with backward selection

^a^Incorporates weighted scores for 19 medical conditions, higher scores indicating worse history of disease

**Fig. 1:**
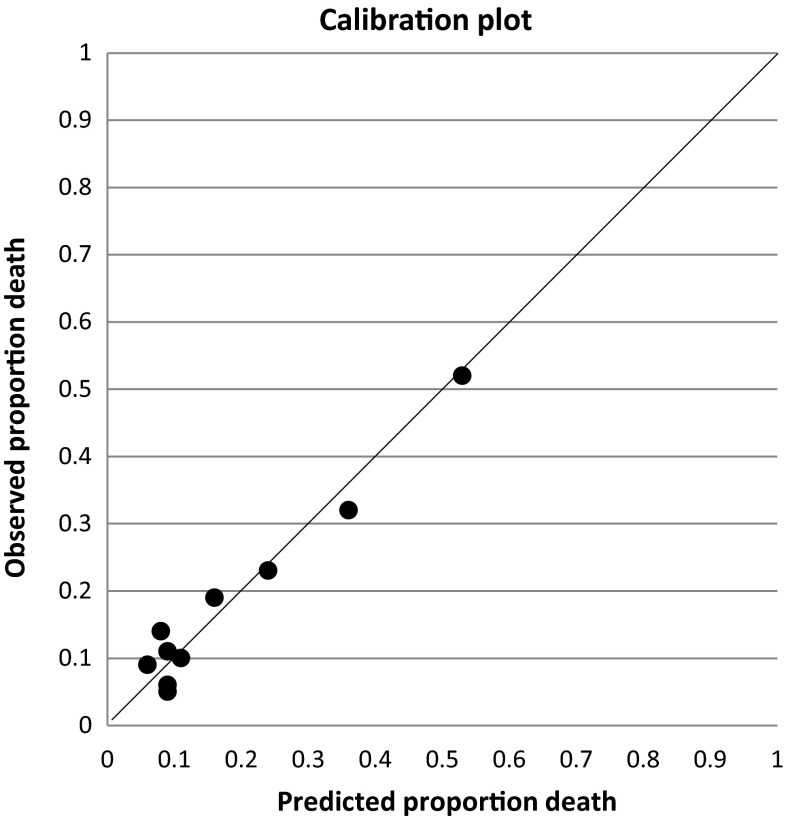
Comparison of observed and predicted 90-day mortality for acutely hospitalized patients into 10 equal groups

By applying the formula 1/(1 + exp(−(−2.127 + 0.862 × ‘saturation’ + 1.201 × ‘CCI’ + 0.774 × ‘thrombocytes’ + 0.706 × ‘urea’ + 0.588 × ‘CRP’ + −0.791 × ‘non-fasted glucose’ ))) the individual risk of 90-day mortality in acutely hospitalized older patients was calculated. Figure 1 shows the calibration plot of the final model. Over the whole range predicted probabilities are in line with the observed, with more spread in the higher risk groups. In Table 4 we calculated predictive performance of the 30, 20 and 10 % patients at highest risk to provide information about clinical usefulness. Positive likelihood ratio’s range from 2.70 to 5.06. The positive predicting value of the 51 patients (10 %) at highest risk is 0.53 and implies that 53 % die within 90 days after admission.

**Table 4:** Performance of predicted high risk deciles in older hospitalized patients

	Number of patients	Sens	Spec	PPV	NPV	LR+	LR−
30 % high risk	160	0.64	0.76	0.38	0.90	2.70	0.47
20 % high risk	106	0.50	0.86	0.44	0.89	3.58	0.58
10 % high risk	51	0.29	0.94	0.53	0.86	5.06	0.76

*Sens* sensitivity, *Spec* specificity, *PPV* positive predicting value, *NPV* negative predicting value, *LR+* positive likelihood ratio, *LR−* negative likelihood ratio

As a sensitivity analysis we repeated analyses for the multivariate and final model with continuous data. Accuracy is comparable in both multivariate and final model. The AUC for continuous data is 0.771 (95 %CI 0.717–0.825) and after dichotomization 0.758 (95 %CI 0.702–0.815) in the multivariate model and 0.736 (95 %CI 0.677–0.795) and 0.738 (95 %CI 0.678–0.798) in the final model (data not shown).

## Discussion

In the present study, we developed a prediction model for 90-day mortality in acutely hospitalized older patients using routinely collected clinical parameters describing disease severity and geriatric factors. With this model we are able to identify a high-risk group with an average 53 % risk of mortality within 90 days after admission compared to the baseline risk of 18.2 %.

We developed and internally validated a prediction model for 90-day mortality in acutely hospitalized older patients using six routinely collected clinical parameters and with adding age and gender. Underlying vulnerability of older patients is reflected in the Charlson comorbidity index (CCI) and severity of disease in oxygen saturation, thrombocytes, urea, C-reactive protein and non-fasted glucose. CCI was developed to predict mortality in medical patients [14], and was recently validated in acutely hospitalized older adults to predict both short- and long-term mortality [11]. Models using vital signs have also been previously studied to predict mortality. The acute physiology and chronic health evaluation (APACHE II) is a severity of disease classification system developed to predict in-hospital mortality in intensive care unit patients of all ages [15]. The APACHE II comprises a combination of vital parameters and different laboratory results. The simple clinical score (SCS) is a prediction model for 30-day mortality in acutely admitted patients [8], and consists of 16 parameters, including vital parameters and presentation signs such as new stroke, coma and abnormal ECG. The Modified Early Warning Score (MEWS) was originally designed for the ED setting to identify medical patients at risk of catastrophic deterioration, and was subsequently validated for prediction of in-hospital mortality in hospitalized patients [7]. The MEWS incorporates five vital signs: systolic blood pressure, pulse rate, respiratory rate, body temperature and level of consciousness. The aforementioned models are well validated and are used in practice, but share the disadvantage that prognostic accuracy among older patients is modest with relatively low positive predicting values. An explanation might be the use of (bed-side) scores with a cut-off point, instead of using individual risk scores. Or it could be the use of either severity of disease characteristics or geriatric factors in the prediction model. Another explanation could be that prediction models were developed in a more severely ill population of all ages, with the consequence that results were neither representative nor tailored for these older patients [16, 17]. Unexpected findings is the positiveness of abnormal thrombocytes and urea. To our knowledge, these measurements are not used in other comparable prediction models. Validity of this might be explained by the possible over-representation of patients with low thrombocytes being treated with chemotherapy or high urea caused by dehydration or kidney failure. Another unexpected finding is the protective value of creatinine clearance <30 (ml/min/1.73m^2^) on 90-day mortality (OR 0.48, 95 % CI 0.28–0.84) in the univariate analysis. A possible explanation could be that the hospital is a centre for patients requiring dialysis and kidney transplantation. These patient groups are hospitalized more readily, with possible less severe acute medical conditions. However, in the multivariate model and by using creatinine clearance as a continuous variable the association is lost, indicating that it could also be caused by outliers. Taken together, we show that combining parameters reflecting severity of disease and geriatric factors results in an prediction model capable of predicting 90-day mortality in acutely hospitalized older patients.

We made several choices in developing our model, in order to be ready for clinical implementation. First, we used routinely available clinical parameters. Candidate predictors were known within 24 h after admission. Second, we used a formula instead of a bed-side scorecard. Health care professionals do not have to calculate risks by hand, preferable the formula should be integrated in the electronic patients records or be available in a smartphone application. By providing an individual risk for each patient, the consequences of the screening can vary. Depending on the local resource availability a hospital can implement interventions. As an example, comprehensive geriatric assessment could be performed in all older patients with a risk of 30 % or higher, which includes extra attention for patient preferences, treatment goals and possible palliative trajectories. In another hospital the advice for the treating physician could be to take into account both the individual risk score and the condition of the patient in decision making, without standardized interventions. Third, we aimed for a model with a high specificity, resulting in a high positive predictive value (PPV) in relation with the baseline risk. The PPV will give the clinician a robust feeling and may therefore be more relevant than a high AUC with modest predictive values. The identification senior at risk (ISAR) is also a prediction tool, originally developed to identify older patients in the ED at increased risk of adverse health outcomes, a composite outcome of functional decline and mortality [18]. The ISAR is a widely used tool in the ED [19], and validated among 667 acute hospitalized older adults for prediction of adverse outcomes, including mortality. After 90 days of follow-up 5 % had died, with 6 % of the patients assigned high risk deceased within 90 days, indicating a low positive predictive value. The negative predictive value (NPV) for 90-day mortality was 0.97, which means that 97 % of the patients not at risk were still alive after 90 days. These results imply that the ISAR in this setting is more suitable to rule out patients at high risk, whereas our model is tailored to identify older patients at high risk for mortality with a PPV of 0.53 in the highest risk group. Identifying of patients at low risk (“rule-out”) may be a very sensible strategy in its own right. However, our aim is to specifically identify patients at the highest risk because these are the patients we want to follow-up with intervention, and we want to aim our limited clinical resources to only those at the highest risk.

Our study has several limitations. First, we studied retrospective data, and therefore the number of available predictors and related outcomes were limited. Ideally, predictors such as cognition, functional status and outcomes such as functional decline, and readmissions should also be used, but these were not available in this retrospective study. Second, the fact that we found some unexpected results further stresses the need for external validation, as it is impossible to distinguish whether these findings are specific to our cohort, chance finding or reproducible in other cohorts. Strengths of the present study are that our prediction model is based on routinely measured and directly available candidate predictors. This enhances convenient future implementation in an early phase of presentation. We used clinical cut-off points to reflect clinical practice and relate to the awareness of the physician. From a methodological point of view using continuous variables is preferable, but is harder to relate to clinical practice. Nevertheless, accuracy of our model is equally well when dichotomized or with continuous variables. Another strength is the high specificity of the developed model. This specificity ensures the development of interventions that are aimed at a relatively small group of patients at high risk of a negative event. Such tools are of importance in the emergency medicine setting, allowing physicians in EDs and Acute Wards to make informed decisions on diagnostic and therapeutic strategies in older patients and the implementation of measures to prevent poor outcome.

In conclusion, we developed a prediction model on 90-day mortality in acutely hospitalized older patients. We used a combination of predictors containing information about severity of disease and geriatric factors and calculated individual risk scores. Currently, we are conducting a large multicentre prospective follow-up study among acutely presenting older patients in both the ED and wards, including more candidate predictors and outcomes.

## Electronic supplementary material

Below is the link to the electronic supplementary material.

Supplementary material 1 (DOCX 68 kb)
