# Supplementary material for: Predicting mortality in acutely hospitalized older patients: a retrospective cohort study
Source: Intern Emerg Med. 2016 Jan 29;11:587–94. doi: 10.1007/s11739-015-1381-7 (PMC4853459; doi:10.1007/s11739-015-1381-7)

**Appendices**

*Manuscript:*

**Predicting mortality in acutely hospitalized older patients; a retrospective follow-up study.**

J. de Gelder,MD^1^; J.A. Lucke,MD^2^; N. Heim, PhD^1^; A.J.M. Craen, PhD^1^; S.D. Lourens, MD^1^; E.W. Steyerberg, PhD^3^; B. de Groot, MD, PhD ^2^; A.J. Fogteloo, MD, PhD^4^; G.J. Blauw, MD, PhD^1^; S.P. Mooijaart, MD, PhD^1,5^

1. Department of Gerontology and Geriatrics, Leiden University Medical Center, the Netherlands
2. Department of Emergency Medicine, Leiden University Medical Center, the Netherlands
3. Department of Public Health, Erasmus MC, Rotterdam, the Netherlands
4. Department of Internal Medicine, section acute care, Leiden University Medical Center, the Netherlands
5. Institute of Evidence-Based Medicine in Old Age | IEMO, Leiden, the Netherlands

Corresponding author

Jelle de Gelder, MD

Leiden University Medical Center

Dept. Gerontology and Geriatrics

PO Box 9600

2300 RC Leiden

The Netherlands

Phone: +31 71 526 6640

Fax: +31 71 524 8945

**Table**

| Supplemental table 1: Overview of imputation and dichotomization of predictors in the study population. | | | | | |
| --- | --- | --- | --- | --- | --- |
| **Predictor** | **Number imputed** | **Unit** | **Clinical**  **reference range** | **Selected reference range** | **N (%) in selected reference range (%)** |
| Saturation | 9 | Percentage | - | **≥96%** | 409 (79.1%) |
| Systolic blood pressure | 0 | mm Hg | 100-140 | **101-199** | 478 (92.5%) |
| Diastolic blood pressure | 0 | mm Hg | 60-80 | **60-89** | 311 (60.2%) |
| Heart rate | 0 | rates/min | 60-99 | **60-99** | 381 (61.5%) |
| Charlson Comorbidity Index^a^ | 0 | score | **-** | **0-4** | 426 (82.4%) |
| Number of medications | 0 | - | ≥ 5: polypharmacy | **0-9** | 364 (70.4%) |
| Thrombocytes | 58 | x 10^9^/L | 150-400 | **150-400** | 378 (73.1%) |
| Urea | 15 | mmol/L | 2.5-7.5 | **≤14.9** | 391 (75.6%) |
| Leukocytes | 14 | x 10^9^/L | 4.00-10.00 | **4.1-12.9** | 348 (67.3%) |
| Sodium | 13 | mmol/L | 136-144 | **136-144** | 355 (68.7%) |
| Potassium | 13 | mmol/L | 3.6-4.8 | **3.6-4.8** | 383 (74.1%) |
| Haemoglobin | 14 | mmol/L | F: 7.5-10 M:8.5-11 | **F: ≥6.5 M: ≥7.5** | 356 (68.9%) |
| C-reactive protein | 42 | mg/L | 0-10 | **≤99** | 376 (72.7%) |
| Non-fasted glucose | 50 | mmol/L | 3.1-6.4 | **≤6.4** | 156 (30.2%) |
| eGFR | 12 | ml/min/1,73m^2^ | <60 | **≥30** | 441 (85.3%) |
| Abbreviations: eGFR = Estimated Glomerular Filtration Rate a) Incorporates weighted scores for 19 medical conditions, with higher scores indicating worse history of disease. | | | | | |

**Graphics (Graphpad Prism software)**

Appendix Figure 1: Kaplan-Meier survival curve


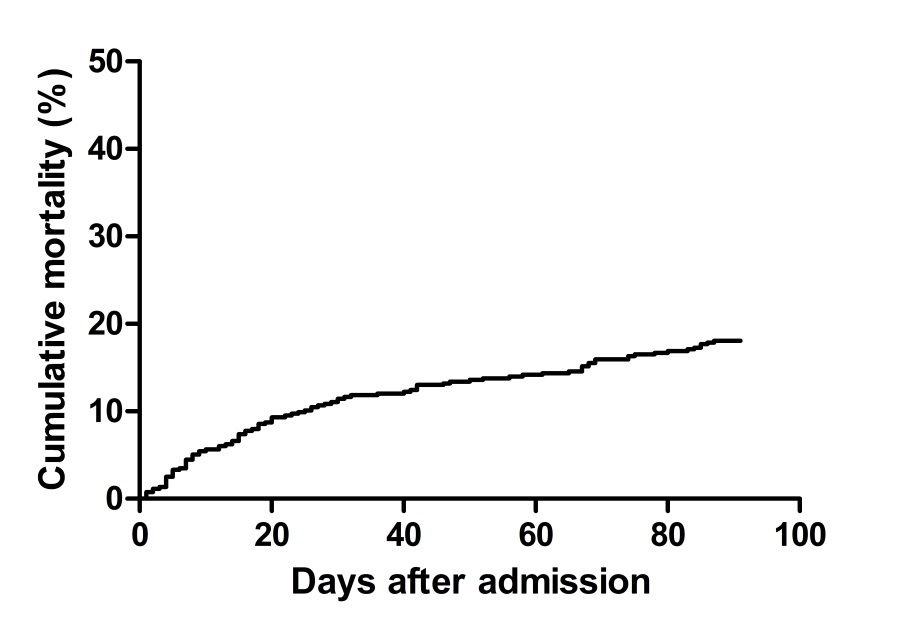

Supplement: Supplementary file 1 — Supplementary material 1 (DOCX 68 kb) [file 11739_2015_1381_MOESM1_ESM.docx]
